# A Comparative Analysis Between Ketamine Versus Combination of Midazolam and Haloperidol for Rapid Safe Control of Agitated Patients in Emergency Department: A Systematic Review

**DOI:** 10.7759/cureus.26162

**Published:** 2022-06-21

**Authors:** Hany A Zaki, Eman Shaban, Khalid Bashir, Haris Iftikhar, Adel Zahran, Emad El-Din M Salem, Amr Elmoheen

**Affiliations:** 1 Emergency Medicine, Hamad Medical Corporation, Doha, QAT; 2 Cardiology, Al Jufairi Diagnosis and Treatment, Doha, QAT; 3 Medicine, Qatar University, Doha, QAT; 4 Anesthesia, Alexandria Armed Forces Hospital, Alexandria, EGY

**Keywords:** efficacy, agitated patients, benzodiazepine, haloperidol, ketamine

## Abstract

We aim to discuss the efficacy and adverse effects of using ketamine in agitated patients in the emergency department (ED) compared with the combination therapy of haloperidol with benzodiazepine.

This systematic review followed Preferred Reporting Items for Systematic Review and Meta-analyses (PRISMA) guidelines. An electronic search from PubMed/Medline, Cochrane library, and Google Scholar was conducted from their inception to 30^th^ April 2022. We included agitated patients in ED who were given infusion with ketamine only. Our comparative group was patients infused with combined therapy of haloperidol and benzodiazepine. We did not include letters, case reports, abstracts, conference papers, appraisals, reviews, and studies where full text was unavailable. We did not put any language restrictions.

Three studies were selected in our manuscript (one cohort and two randomized controlled trials). All three studies showed that ketamine was used to achieve sedation in less time than the other group. However, two studies reported significantly more adverse effects in ketamine-infused groups.

We concluded that ketamine use is superior when its primary focus is to sedate the patient as quickly as possible, but it carries some side effects that should be considered. However, we still need more studies assessing the efficacy of ketamine in agitated patients presenting in the ED.

## Introduction and background

Prehospital providers are often faced with the challenge of carefully bringing acutely agitated patients with psychiatric or organic pathologies to clinicians in the emergency department (ED) [1]. Combative patients with severe psychomotor agitation pose a physical threat to themselves and the staff. Physical restraints were used to control the violent behaviors of agitated patients [1]. However, complications like asphyxia, hyperkalemia, and excited delirium syndrome have made the use of chemical restraints more appealing to physicians [2]. Failure to respond to verbal de-escalation, which is often the first step in managing such patients, is followed by the use of parenteral medication in agitated patients [3].

Benzodiazepines like midazolam and lorazepam are common sedative drugs used in the emergency setting [4]. Benzodiazepines bind to the α and γ subunits of the gamma-aminobutyric acid (GABA) receptor commonly found in the cortex and limbic system [4]. They are used for anti-anxiolytic, amnestic, hypnotic, and sedative effects. Midazolam is a short-acting agent, available via intranasal (IN), intravenous (IV), and intramuscular (IM) routes for rapid sedation [5]. The average time required for sedation after a recommended dose of 2-5 mg IM ranges from 13 to 18 minutes [6]. Lorazepam is administered via IV, IM, or IN route for rapid sedation [5,6]. In the ED, dosing ranges from 0.5 to 2 mg IM or IV. The time required for adequate sedation with lorazepam is around 32 minutes [6].

First-generation antipsychotics are helpful for sedation in alcohol-intoxicated patients. Haloperidol is a first-generation antipsychotic that blocks D2 dopamine receptors and is administered via oral, IV, or IM routes [5,6]. Sedation is achieved in 25-28 minutes after a dose of 2.5-10 mg [6]. The major risks associated with haloperidol include extrapyramidal side effects such as akathisia and dystonia. It is best reserved for patients with alcohol intoxication.

Ketamine is an anesthetic agent similar to phencyclidine (PCP), which works by inhibiting N-methyl-D-aspartate (NMDA) receptors and stimulating opioid receptors [7]. Patients with severe hypotension or respiratory depression benefit mostly from the use of ketamine for inducing sedation. With IM administration, the time for sedation is around 4-5 minutes. IV route has a much more rapid onset of action (1-2 minutes) [7]. Recommended dosing ranges from 1 to 2 mg/kg IV and 4 to 6 mg/kg IM [7]. One study compared ketamine to haloperidol for the time required for sedation in a prehospital setting. Ketamine was significantly better than haloperidol (5 minutes vs 17 minutes) in the prehospital setting [8]. However, 49% of patients in the ketamine group experienced complications like akathisia, hypersalivation, and vomiting as compared to 5% of patients in the haloperidol group [8].

Nobay et al. compared time to achieve sedation following intramuscular infusion of haloperidol, lorazepam, and midazolam in agitated patients in a prehospital setting [9]. Midazolam was significantly better (p < 0.05) than haloperidol and lorazepam in time to onset of sedation and time to arousal [9]. Another study compared haloperidol and midazolam in agitated patients in a prehospital setting for achieving sedation. Midazolam required 13.5 minutes to reach a Richmond agitation sedation scale (RASS) score of less than +1, and haloperidol required 24 minutes [10].

## Review

Method

Data Sources and Search Strategy

This systematic review was performed by following Preferred Reporting Items for Systematic Review and Meta-analyses (PRISMA) guidelines [11]. An electronic search from PubMed/Medline, Cochrane library, and Google Scholar was conducted from their inception to 30th April 2022 using the search string (Ketamine OR ketamine hydrochloride) AND (midazolam OR benzodiazepine) AND (haloperidol OR Haldol). Furthermore, we manually screened the cited articles from previous meta-analyses, systematic reviews, cohort studies including retrospective or prospective studies, and other review articles to identify any relevant studies.

Study Selection

All studies were included if they met the following eligibility criteria, which can be given as PICOS - (1) P (Population): agitated people presenting in ED; (2) I (Intervention): ketamine infusion as a single agent; (3) C (Comparison): combined effect of haloperidol and benzodiazepine; (4) O (Outcome): a comparative analysis between the intervention group and control group, and (5) S (Studies): randomized controlled trials published in English.

We excluded letters, case reports, abstracts, conference papers, appraisals, reviews, and studies where full text was unavailable. Only English-based articles were included. We excluded if ketamine was used for a different indication or outside of the ICU or ED. We exclude all studies using ketamine in combination with other drugs.

Data Extraction and Quality Assessment of Studies

Two reviewers independently searched electronic databases. Studies searched were exported to the EndNote Reference Library software version 20.0.1 (Clarivate Analytics), and duplicates were screened and removed.

Data extraction and quality assessment of included studies were done simultaneously and independently by three reviewers. Newcastle-Ottawa scale (NOS) was used to assess the quality of the cohort studies. NOS score < 6 was considered high risk for bias, 6-7 was moderate, and a score >7 was considered a low risk of bias (Table 1).

**Table 1:** Quality assessment of the cohort study using the new Ottawa scale

Study	Selection (Maximum 4)				Comparability (Maximum 2)	Outcome (Maximum 3)			Total score
	Representativeness of the exposed cohort	Selection of the non-exposed cohort	Ascertainment of exposure	Demonstration that the outcome of interest was not present at the start of the study	Comparability of cohorts on the basis of the design or analysis	Assessment of outcomes	Was follow-up long enough for outcomes to occur	Adequacy of follow-up of cohorts	
O’Connor et al., 2019 [12]	1	1	1	1	2	1	1	1	9

The risk of biases from randomized control trials (RCTs) was assessed through Cochrane Collaboration’s tool in seven domains: adequate sequence generation, allocation concealment, blinding of participants and personnel, blinding of outcome assessment, incomplete outcome data, and selective outcome reporting, free of other bias. The individual domains and overall risk-of-bias judgment were expressed on one of three levels: low risk of bias, unclear risk of bias, and high risk of bias. Based on these factors, the overall quality of evidence was deemed low, moderate, or high risk of bias (Table 2).

**Table 2:** Quality assessment of randomized controlled trials using Cochrane Collaboration’s tool

Study	Random sequence generation	Allocation concealment	Blinding (participants and personnel)	Blinding (outcome assessment)	Incomplete outcome data	Selective reporting	Other sources of bias	Net risk
Lin et al., 2021 [13]	Low risk	Low risk	High risk	High risk	Low risk	Low risk	Low risk	Low risk
Barbic et al., 2021 [14]	Low risk	Low risk	Low risk	Low risk	Low risk	Low risk	Unclear risk	Low risk

Statistical Analysis

The results were formed using a qualitative analysis. We intended to summarize the findings of the articles to synthesize the results. It was selected as it incorporated the results from all articles and highlighted all the differences and similarities between the study findings. Since the included studies showed heterogeneity among their evaluation criteria and study results, with not all studies containing appropriate findings to carry out a meta-analysis, we decided to do only a systematic review instead.

Results

Literature Search Results

**Figure 1:**
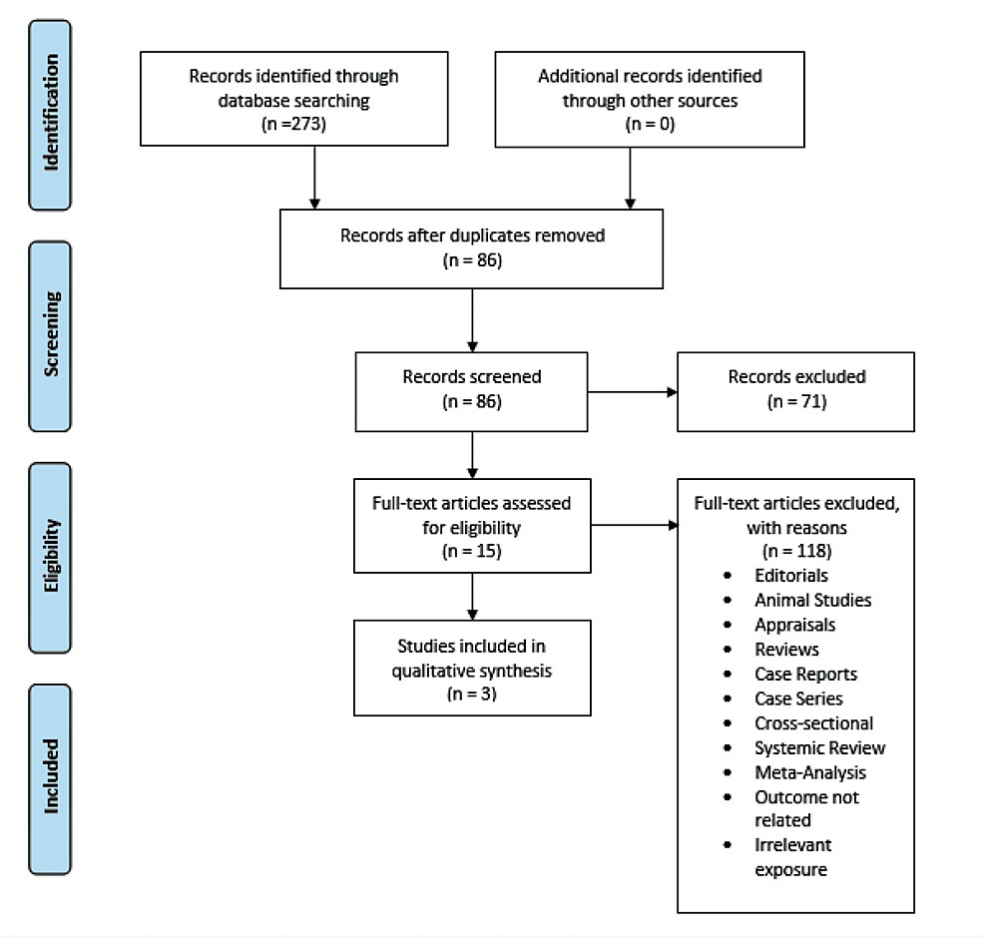
PRISMA flow diagram of the literature search results PRISMA: Preferred Reporting Items for Systematic Reviews and Meta-Analysis.

The initial search of the three electronic databases yielded 273 potential studies. After exclusions based on titles and abstracts, the full texts of 15 studies were read for possible inclusion. A total of three studies remained for qualitative analysis. Figure 1 summarizes the results of our literature search.

Study Characteristics

Table 3 provides the essential characteristics of included studies. We had one cohort and two RCTs. This systematic review used 328 patients. Two studies were conducted in the United States and one in Canada. The mean age was 40.07 years. Further notes on these studies are provided in Table 3.

**Table 3:** Characteristics of selected studies T: Total number of patients; I: Patients in the intervention group; C: Patients in the control group; RCT: Randomized control trial; USA: United States of America; IM: Intramuscular; IV: Intravenous; ED: Emergency Department.

Study	Year	Study design	Duration	Country	Total patients (n)	Mean age (years)	Dose of ketamine	Dose of haloperidol	Dose of benzodiazepine	Net risk of bias	Other notes
O’Connor et al. [12]	2019	Cohort	Jan 2014-Feb 2018	USA	163 (T) 95 (I) 68 (C)	34.9 (77.7)	3.68 mg/kg IM	5 mg IM	2-4 mg IM of midazolam	Low risk	Higher intubation rates and use of additional chemical restraint were seen with ketamine use when compared with the haloperidol + benzodiazepine group. ED length of stay was the same in both groups.
Lin et al. [13]	2021	RCT	Jan 2018-Oct 2018	USA	86 (T) 41 (I) 45 (C)	50 (54.07)	4 mg/kg IM or 1 mg/kg IV	10 mg IM or IV	2 mg IM or IV of lorazepam	Low risk	The ketamine group achieved sedation in a median time of 15 minutes as compared to 36 minutes for the haloperidol + lorazepam group.
Barbic et al. [14]	2021	RCT	June 2018-March 2020	Canada	79 (T) 39 (I) 40 (C)	35.3 (9.25)	5 mg/kg IM	5 mg IM	5 mg IM of midazolam	Low risk	The median time to sedation was significantly shorter for ketamine (5.8 minutes) as compared to midazolam + haloperidol (14.7 minutes).

Quality Assessment

All studies have a low risk of bias (Tables 1, 2).

Results of Qualitative Analysis

We included three studies in our manuscript [12-14]. O’Connor et al. was a retrospective cohort study that evaluated the effect of ketamine, a combination of haloperidol and benzodiazepine, and physical strain on agitated patients who came to ED [12]. While the other two studies by Lin et al. and Barbic et al. were RCTs that conducted a comparative parallel study to assess the efficacy between the use of ketamine only and a combination dose of haloperidol and benzodiazepine [13,14].

Efficacy

We assessed the efficacy based on which intervention was more sedative in nature. All studies reported that ketamine was a significantly more effective sedative than the combination of haloperidol and benzodiazepine. It was observed that more time was taken by the combination therapy to complete sedation. Lin et al. reported that it took a median time of 15 minutes to achieve sedation in ketamine, while a median time of 36 minutes was seen in the combined therapy (p < 0.001) [13]. Similarly, Barbic et al. showed a median difference of 8.8 minutes for sedation to take place between the two intervention groups, whereas ketamine achieved sedation more quickly [14]. In addition, O’Connor et al. reported that fewer additional interventions were needed to sedate patients on ketamine as compared to the combination therapy (OR = 2.94 [95% CI, 1.49-5.80]) [12].

Adverse Effects

In two studies, Lin et al. and Barbic et al. reported the adverse effects of using ketamine as single infusion therapy and combining haloperidol with benzodiazepine to relax agitated patients in ED. Both the studies showed that relatively more adverse effects occurred after ketamine use rather than the combination therapy [13,14]. Lin et al. reported significant adverse effects of hypertension (p = 0.01) and tachycardia (p = 0.01) in ketamine group [13]. Barbic et al. also showed more episodes of adverse effects in patients who were given ketamine than the combination therapy (12.5% of patients vs 5% of patients, respectively) [14].

Discussion

In this systematic review, we evaluate the effectiveness of ketamine in comparison to the combined therapy of midazolam and haloperidol. The evidence from three included studies suggested that ketamine showed superior efficacy in terms of sedation; however, ketamine was associated with more adverse events than the combined therapy.

The traditional first-line therapy for severely agitated ED patients is a benzodiazepine, haloperidol, or both [15]. However, despite the wide use of benzodiazepine and antipsychotics, they have inadequate sedative effects [16]. In contrast, ketamine has gained attention as an alternative short-acting sedative [17]. Several studies showed that ketamine exerts adequate sedative effects within 5 minutes in 90%-96% of recipients who receive 4-5 mg/kg IM ketamine [15,18,19]. In contrast, 10 mg IM haloperidol requires a median of 17 minutes to achieve sedation; however, only 65% of the recipients were adequately sedated [15,19]. Ketamine was also associated with less redosing of sedative agents compared to haloperidol (5%-20%) [15]. Ketamine PCP analog results in non-competitive NMDA antagonism, thereby decreasing the calcium influx and ultimately blocking the neuronal activation required for the conscious state [20]. The evidence from the literature also suggested the interaction of ketamine with other receptors, such as opioid, serotonin, dopamine, muscarinic, and adrenergic receptors [21,22].

Ng et al. assessed the role of ketamine in agitated children in the ED. They reported that ketamine was associated with a lesser incidence of emergence agitation and a low postoperative pain score. No adverse effect was reported. However, the results were inconclusive because of the low certainty of evidence [23]. Bak et al., in their systematic review and meta-analysis, found that olanzapine, haloperidol, plus promethazine or droperidol are the most effective and safer intervention in the treatment of agitation [24]. Several other meta-analyses and systematic reviews have been published on this subject; however, in our literature, we could not find any previously published systematic reviews on this topic [25-27].

Ketamine has a well-known adverse event profile. The common adverse effects include nausea, vomiting, tachycardia, hypertension, hypersalivation, emergence reaction, and laryngospasm. However, the adverse effects are more pronounced with the rapid IV administration and less prominent with slower delivery [28]. In contrast, benzodiazepine and haloperidol result in extrapyramidal symptoms, respiratory depression, and long QT syndrome. Respiratory depression is a particular concern for benzodiazepines [15].

In this qualitative analysis, two studies presented adverse events associated with the interventions. Lin et al. reported significant hypertension and tachycardias associated with ketamine; however, the adverse events were resolved before leaving the ED in a majority of patients. Statistically, non-significant hypoxia was reported in ketamine recipients. One patient in the haloperidol and benzodiazepine group experienced bradycardia, hypoxia, cardiac arrest, and subsequent death. Similarly, Barbic et al. reported apnea (n = 2), supplementary oxygen requirement (n = 1), laryngospasm (n = 1), and dystonia (n = 1) in ketamine group. However, two patients in the haloperidol and midazolam group had adverse events, apnea (n = 1), and supplementary oxygen requirement (n = 1).

The results of our study suggested that ketamine showed superior sedative effects in comparison to the combined administration of midazolam and haloperidol. However, ketamine was associated with more pronounced adverse effects, predominantly hypertension, tachycardia, and hypoxia. Despite that, in the prehospital setting, time from administration to adequate sedation is a priority, therefore making rapid-acting ketamine an appealing option.

Limitations

Our study had the following limitations: (a) minimum studies with a small sample size were used qualitatively to analyze the findings; (b) findings of one cohort were discussed with two RCTs which might cause some bias, and (c) this analysis discussed studies only from the United States and Canada (representing the same region). Despite these shortcomings, these studies were pivotal in assessing the importance of ketamine infusion in agitated patients presenting in ED compared with combined therapy of haloperidol with benzodiazepine.

## Conclusions

The results of the qualitative analysis suggested that ketamine showed a superior and rapid sedative effect in agitated patients in the ED, compared to combining therapy with midazolam and haloperidol. Ketamine was associated with shorter sedative effects, and less redosing of sedative agents is required in ketamine-induced sedation in comparison to haloperidol.

Statistically significant adverse events were reported in the ketamine group; tachycardia, hypertension, and hypoxia were more pronounced and resolved spontaneously or with minor interventions. Adverse effects such as bradycardia, hypoxia, cardiac arrest, and apnea were reported in patients in midazolam and haloperidol group. In the prehospital setting, time from administration to adequate sedation is a priority, therefore making rapid-acting ketamine an appealing option. However, the presented evidence is not sufficient enough to predict stronger results. Larger trials should be conducted on this subject to evaluate the role of ketamine in comparison to midazolam and haloperidol on severely agitated patients in the ED.
